# Impact of systemic lupus erythematosus on cardiovascular morphologic and functional phenotypes: a Mendelian randomization analysis

**DOI:** 10.3389/fcvm.2024.1454645

**Published:** 2024-10-03

**Authors:** Zishan Lin, Wenfeng Wang, Bingjing Jiang, Jian He, Yanfang Xu

**Affiliations:** ^1^ Department of Nephrology, Blood Purification Research Center, The First Affiliated Hospital, Fujian Medical University, Fuzhou, China; ^2^ Research Center for Metabolic Chronic Kidney Disease, The First Affiliated Hospital, Fujian Medical University, Fuzhou, China; ^3^ Department of Nephrology, National Regional Medical Center, Binhai Campus of the First Affiliated Hospital, Fujian Medical University, Fuzhou, China; ^4^ Department of Gastroenterology, Nanfang Hospital, Southern Medical University, Guangzhou, China

**Keywords:** systemic lupus erythematosus, cardiovascular magnetic resonance imaging, cardiovascular structure and function, Mendelian randomization, cardiac function

## Abstract

**Background:**

Previous studies have established a correlation between systemic lupus erythematosus (SLE) and cardiovascular health, but the potential causal effects of SLE on heart function and structure remain poorly understood. Cardiovascular magnetic resonance imaging (CMR), a novel non-invasive technique, provides a unique assessment of cardiovascular structure and function, making it an essential tool for evaluating the risk of heart disease. In this study, we performed a Mendelian randomization analysis to determine the causal relationship between SLE and CMR traits.

**Methods:**

Genetic variants independently linked to SLE were selected from a genome-wide association study (GWAS) containing 5,201 cases and 9,066 controls as instrumental variables. A set of 82 CMR traits was obtained from a recent GWAS, serving as preclinical indicators and providing preliminary insights into the morphology and function of the four cardiac chambers and two aortic segments. Primary analysis employed a two-sample Mendelian randomization study using the inverse-variance weighted method. Heterogeneity testing, sensitivity analyses, and instrumental variable strength assessments confirmed the robustness of the findings.

**Results:**

SLE exhibited a correlation with increased stroke volume (β_LVSV_ = 0.007, *P* = 0.045), regional peak circumferential strain (β_Ecc_AHA_9_ = 0.013, *P* = 0.002; β_Ecc_AHA_12_ = 0.009, *P* = 0.043; β_Ecc_AHA_14_ = 0.013, *P* = 0.006), and global peak circumferential strain of the LV (β_Ecc_global_ = 0.010, *P* = 0.022), as well as decreased regional radial strain (β_Err_AHA_11_ = −0.010, *P* = 0.017).

**Conclusions:**

This research presents evidence of a potential causal association between traits of SLE and alterations in cardiac function, guiding cardiac examinations and disease prevention in lupus patients.

## 1 Introduction

Systemic lupus erythematosus (SLE) is a chronic autoimmune disease that commonly affects multiple organs and is associated with high prevalence and mortality. Several observational studies have indicated a relationship between SLE and the development of heart disease, such as cardiovascular diseases, myocarditis, valvular heart diseases, and heart failure (1–4). It is well-documented that structural changes in the heart may precede the onset of cardiac conditions. Notably, individuals with lupus who manifest heart disease often exhibit alterations in heart structure or function in clinical settings (1, 4). These alterations in lupus patients may be associated with the aforementioned cardiac conditions. However, these findings may be influenced by biases arising from residual confounding factors and reverse causality, due to the inherent limitations of observational studies (5). Therefore, caution is advised when interpreting causality in these correlations, and randomized controlled trials or advanced statistical methods are required to validate these findings and minimize biases and confounding factors.

Cardiac and aortic structures, crucial for maintaining normal physiological functions, can manifest abnormalities even before overt disease symptoms present. The utilization of cardiovascular magnetic resonance imaging (CMR) allows for the comprehensive integration of morphological and functional assessments, enabling precise tissue characterization of myocardial changes (6). This capability provides detailed insights comparable to pathological observations of various myocardial abnormalities, including edema, necrosis, fibrosis, and more (7, 8). As a result, CMR is widely recognized as the gold standard for the non-invasive assessment of cardiovascular structure and function. The attributes obtained from CMR serve as recognized endophenotypes, playing a crucial role as key risk indicators (9, 10). The ongoing exploration of gene repositories has facilitated the availability of genome-wide association studies (GWAS) associated with CMR phenotypes, supporting in-depth research enhanced by pertinent GWAS data.

As an evolving epidemiological research approach, Mendelian randomization (MR) analysis employs genetic variants as instrumental variables (IVs) to evaluate the causal relationships between exposures and outcomes. In conventional observational studies, confirming the causal relationship between SLE and heart structure and function presents challenges due to the complexities of reverse causality and residual confounders. In contrast, IVs in MR analysis have unique advantages. Alleles are randomly assigned from parents to offspring according to Mendel’s laws of inheritance, ensuring genetic variation largely independent of confounding factors. Additionally, MR analysis adheres to the natural order of causation, as genetic variation precedes both exposure and outcome (11). Therefore, we conducted a two-sample MR study to examine the potential causal relationships between SLE and these CMR traits.

## 2 Methods

### 2.1 Study design

**Figure 1:**
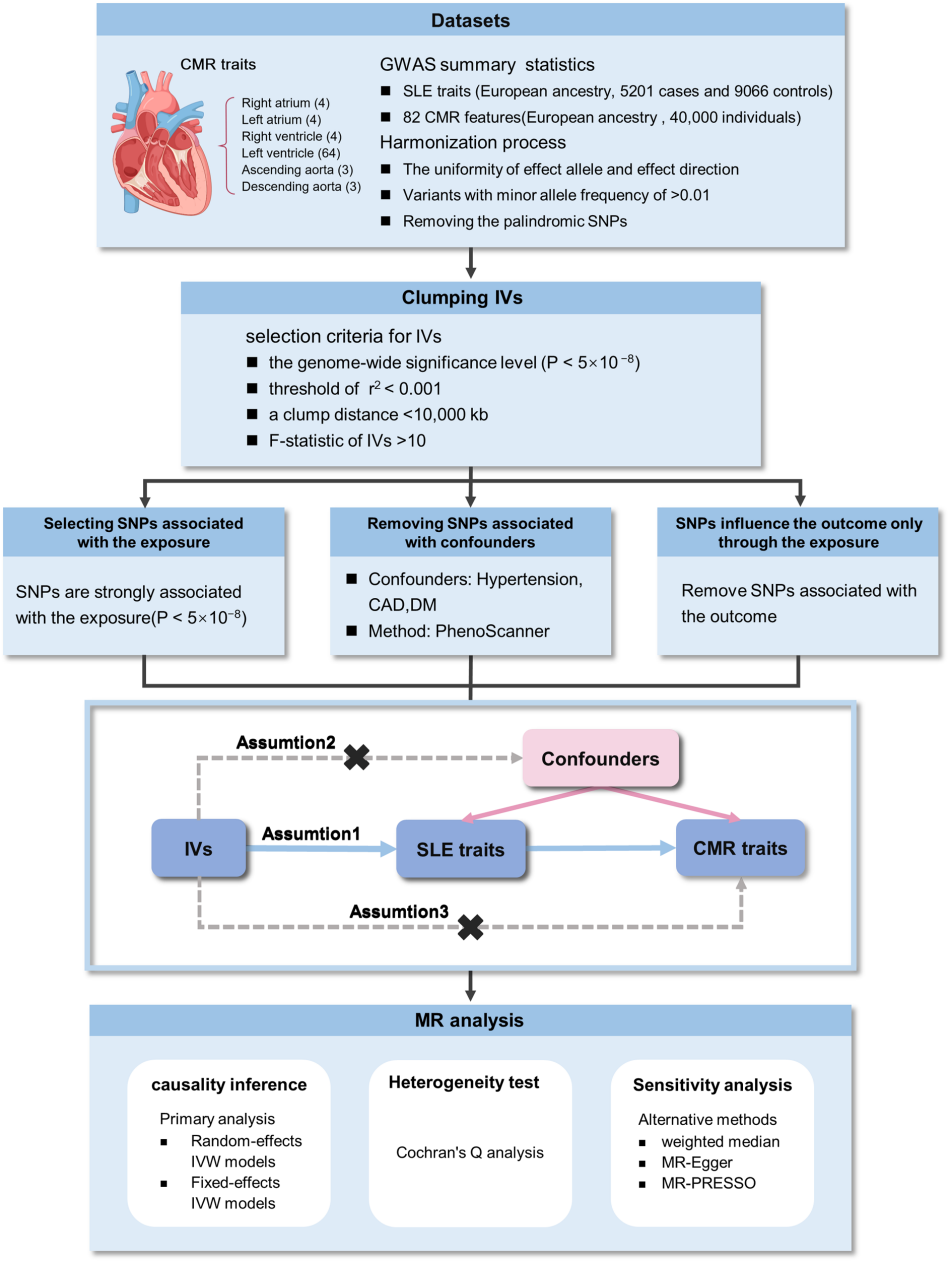
Overview of the study design and analyses. CMR, cardiovascular magnetic resonance imaging; GWAS, genome-wide association studies; SLE, systemic lupus erythematosus; SNPs, single nucleotide polymorphisms; IVW, inverse-variance weighted; IVs, instrumental variables.

Figure 1 displays an overview of the research methodology. A two-sample MR study was conducted to assess causal associations linking SLE and CMR characteristics, with SLE as the exposure and CMR features as the outcomes. This investigation was underpinned by three pivotal assumptions to obtain a persuasive conclusion from MR analysis: (1) IVs demonstrate a robust correlation with SLE; (2) IVs are independent of potential confounders; and (3) IVs predominantly influence outcomes through the exposure pathway. The data utilized were exclusively sourced from the publicly available GWAS catalog, and ethical approvals as well as informed consent were obtained in all original papers.

### 2.2 Data source

The single nucleotide polymorphisms (SNPs) associated with SLE were sourced from the study conducted by Bentham J. et al., which was published in 2015 (12). This investigation involved a newly conducted GWAS, a meta-analysis, and a replication study, encompassing 14,267 individuals of European ancestry (5,201 cases and 9,066 controls). For the outcome datasets, 82 CMR features were primarily sourced from a study conducted by Zhao et al. in 2023, including four left atrium (LA) traits, 64 left ventricle (LV) traits, four right atrium (RA) traits, four right ventricle (RV) traits, three ascending aorta (AAO) traits, and three descending aorta (DAO) traits. This research involved a cohort of 40,000 participants and employed the analytical pipelines developed by Bai et al. for extracting imaging traits from raw brain and cardiac MRI images (13). The datasets for SLE and CMR traits were limited to individuals of European ancestry to minimize potential bias. The summary of the GWAS data used in our study is provided in Table 1. Further details regarding the exposure and results data, such as trait names and categories can be found in Supplementary Table S1, S2.

**Table 1:** Studies and datasets included in the mendelian randomization analyses.

Traits	Data source	Sample size (cases/controls)	Year	Ancestry	PMID
Exposure					
Systemic lupus erythematosus	Bentham J. et al.	5,201/9,066	2015	European	26502338
Outcome					
Cardiovascular magnetic resonance imaging	Zhao et al.	40,000	2023	European	37262162

### 2.3 Genetic instrument selection

The criteria for selecting IVs required SNPs to be significantly correlated with exposures, achieving genome-wide significance (*P* < 5 × 10^−8^). Additionally, linkage disequilibrium clumping procedures were applied with a threshold set at *r*^2^ < 0.001 and a maximum clump distance of <10,000 kb to ensure the independence of SNPs. All of these SNPs were searched for secondary phenotypes using the PhenoScanner tool (http://www.phenoscanner.medschl.cam.ac.uk/).log to exclude potential pleiotropic effects. SNPs corresponding to phenotypes directly related to the outcome or associated with confounders (*P* < 5 × 10^−8^), including cardiovascular disease, hypertension, and diabetes were removed. Only genetic instruments with an F-statistic greater than 10 were retained for further analysis to eliminate weak instruments. To assess the robustness of each genetic instrument, the F-statistic was calculated using the specified formula: $F={\left(\frac{\;{\hat{\beta }}_{X}}{Se({\hat{\beta }}_{X})}\right)}^{2}$ (5).

### 2.4 Statistical analyses

Before initiating the MR analysis, the exposure and outcome datasets were harmonized to remove palindromic SNPs. The primary analytical approach for the MR analysis involved using the inverse-variance weighted (IVW) method, which assumes that all SNPs function as valid IVs to achieve the most accurate estimates. Heterogeneity was tested using Cochran’s Q analysis. A *P*-value greater than 0.05 led to the adoption of the fixed-effects IVW approach under the assumption of homogeneity. Conversely, the random-effects IVW method was employed in scenarios where heterogeneity was detected.

### 2.5 Sensitivity analyses

Additional sensitivity analyses were performed using the weighted median, MR-Egger, and MR-PRESSO. Specifically, the weighted median approach posits that a valid causal inference can still be made if at least 50% of the weights from the IVs are valid. The MR-Egger test provides a valid result even in cases where all IVs are invalid. Although both the weighted median and MR-Egger methods have less statistical power compared to the IVW method, consistent results in the same direction across all three methods strengthen the reliability of the causal estimates from the primary analysis. Furthermore, the MR-PRESSO method was utilized to identify and address horizontal pleiotropy introduced by outlier SNPs, which were subsequently identified, removed, and re-analyzed (5). The results of the study were presented as effect value (β) with 95% confidence interval (CI), setting the threshold for significance at α=0.05. All statistical analyses were carried out using Rstudio (version 4.3.1) with the TwoSampleMR package (version 0.5.8).

## 3 Results

### 3.1 Identifying genetic instruments for SLE traits

Following the predefined screening criteria, 39–41 significant (*P* < 5 × 10^−8^) and independent (*r*^2^ < 0.001, 10,000-kb) SNPs were carefully selected as IVs from the SLE GWAS (Supplementary Table S3). No SNPs directly associated with CMR traits were identified, and SNPs associated with confounds were excluded (Supplementary Table S4). Each F-statistic associated with the instrumental exposure exceeded 10 (ranging from 30 to 461), thereby successfully reducing the influence of weak IVs on the study outcomes.

### 3.2 SLE and CMR traits

**Figure 2:**
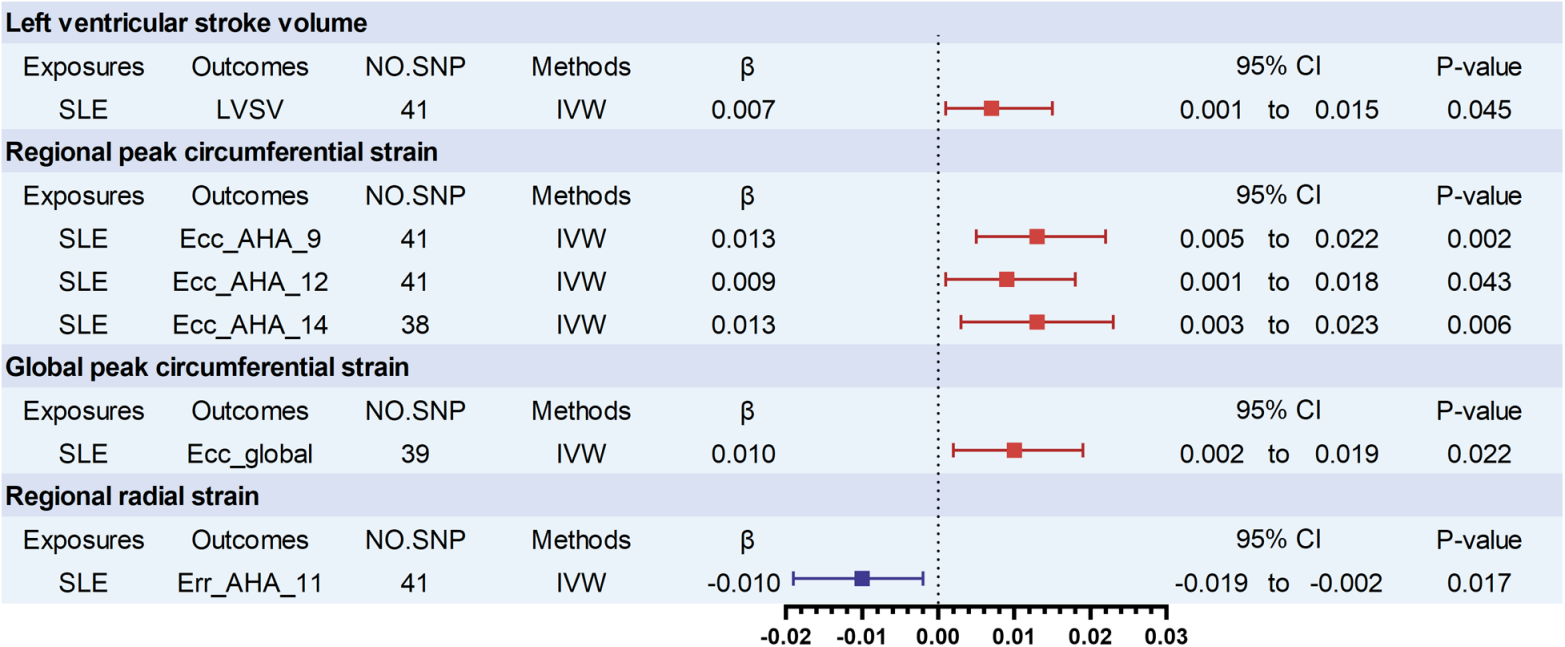
Significant associations of genetically predicted SLE traits with cardiovascular magnetic resonance imaging traits. LVSV, left ventricular stroke volume; Ecc_AHA, regional peak circumferential strain, segments 9, 12, and 14 of the 16 predefined segments by the American Heart Association (AHA); Ecc_global, global peak circumferential strain. Err_AHA, regional radial strain, segment 11 of the 16 predefined segments by the American Heart Association (AHA); SLE, systemic lupus erythematosus.

The main results are presented in Figure 2. In the comprehensive analysis of all outcome data, a genetically predicted SLE exhibited a correlation (*P* < 0.05) with 6 CMR traits. Among these, SLE showed a positive association with left ventricular stroke volume (LVSV) (β_LVSV_ = 0.007, 95% CI 0.001–0.015, *P* = 0.045). Furthermore, SLE was positively correlated with three regional peak circumferential strains of LV from the 16 pre-defined American Heart Association (AHA) segments, as well as global peak circumferential strain. The specific values for each are as follows: Ecc_AHA_9 (β_Ecc_AHA_9_ = 0.013; 95% CI 0.005–0.022; *P* = 0.002), Ecc_AHA_12 (β_Ecc_AHA_12_ = 0.009, 0.001–0.018; *P* = 0.043), Ecc_AHA_14 (β_Ecc_AHA_14_ = 0.013; 95% CI 0.003–0.023; *P* = 0.006), and global peak circumferential strain (β_Ecc_global_ = 0.010, 95% CI 0.002–0.019, *P* = 0.022). In contrast, SLE was negatively associated with a regional radial strain of LV from the 16 pre-defined AHA segments: Err_AHA_11(β_Err_AHA_11_ = −0.010, 95% CI −0.019 to −0.002, *P* = 0.017). There was a notable trend toward significance between SLE and increased right ventricular end-diastolic volume (RVEDV) (β_RVEDV_ = 0.006, *P* = 0.060), although the findings were not statistically significant between SLE and any of AAO, DAO, LA, RA, or RV (Supplementary Table S5).

### 3.3 Sensitivity analyses of MR

Most of the results remained directionally consistent with those from the IVW method, thereby enhancing the robustness of the findings. No statistical associations were found between SLE and CMR traits using the MR-Egger and weighted median methods. No evidence of heterogeneity or horizontal pleiotropy was observed among the statistically significant CMR traits. Some outlier SNPs were identified and removed using the MR-PRESSO method, but their exclusion did not affect the stability of the main findings (Supplementary Table S5).

## 4 Discussion

SLE is a complex autoimmune disorder that can affect various organs, including cardiovascular system. The 2017 European Society of Cardiology Consensus Document by Caforio et al. emphasized the prevalence and severity of cardiac involvement in patients with SLE. The study also highlighted the crucial role of non-invasive diagnostic methods, including CMR, in detecting early cardiac structural changes in these patients (14). Extensive studies have already indicated a possible association between SLE and heart diseases, such as cardiomyopathy, heart failure, valvular heart diseases, and cardiovascular diseases (1–4). Despite advancements in medical technology that have improved the longevity and quality of life for individuals with lupus, mortality linked to cardiovascular events has increased (15, 16). As a major source of early damage in lupus patients, cardiovascular system impairment has garnered considerable scholarly attention in recent years (17). A cohort study involving 252,676 patients with SLE and 758,034 matched controls in the United States revealed an increased risk of CAD linked to SLE (adjusted OR 1.42; 95% CI 1.40–1.44) (18). Furthermore, a long-term observational study of 3,411 SLE patients demonstrated an elevated risk of heart failure and other cardiovascular outcomes in comparison to matched control subjects (19). In 2022, Gao et al. conducted an MR study that confirmed the potential causal relationship between SLE and an elevated risk of heart failure (20). Similarly, Kain et al. leveraged MR and pathway analysis in the same year to identify shared genetic risk factors for SLE and CAD (21). Despite these findings, there remains a gap in research concerning the relationship between lupus and cardiac structure and function, and whether these heart changes observed in lupus patients are solely attributed to SLE is uncertain.

In this investigation, we utilized the latest large-scale GWAS summary-level data to examine the association between SLE and a comprehensive set of 82 CMR traits. These traits were systematically classified into six categories: RA, RV, LA, LV, AAO, and DAO. This study is the first to apply MR to systematically explore the potential causal relationships between SLE and both heart structure and function. The main discovery of our research suggests a potential causal relationship between SLE traits on LV. The findings are summarized as follows: (1) SLE was linked to greater LVSV; (2) SLE was correlated with three increased regional peak circumferential strains from the 16 pre-defined AHA segments (Ecc_AHA_9, 12, 14); (3) SLE was associated with higher global circumferential strain; (4) SLE was related to decreased left ventricular regional radial strain (Err_AHA_11).

In our study, left ventricular function changes associated with SLE were identified. These findings are consistent with several observational studies. For instance, in an observational study of 79 patients with SLE, Myhr et al. identified a higher prevalence of myocardial fibrosis and noted structural changes in the LV compared to the control group. Notably, LVSV was found to be increased in SLE patients compared to normal controls (*P* = 0.03), corroborating our findings (2). As a critical method for evaluating cardiac function, myocardial strain offers detailed insights into myocardial mechanics and functional status (22, 23). A meta-analysis by Di Minno et al. demonstrated a significant reduction in left ventricular radial strain in SLE patients compared to non-SLE controls (95% CI: −13.819 to −8.241, *P* < 0.001), consistent with our study outcomes (24). In this study, we identified an association between SLE and increased left ventricular regional and global peak circumferential strains, which appears to diverge from the findings of previous study (24). It is possible that similar to myocardial injury caused by other etiologies, SLE-induced myocardial fibrosis or endothelial dysfunction varies in severity across different regions of the myocardium. Some areas might initially affect radial strain, while other regions, either unaffected or experiencing compensatory enhancement, could show increased circumferential strain. Therefore, the observed increase in myocardial circumferential strain may reflect a compensatory mechanism of the heart aimed at preserving overall cardiac function. Another possible explanation could be the ethnic differences caused by the fact that our database included only European samples. Larger studies with more diverse populations are needed to determine the association between these factors.

Previous studies have frequently suggested a close association between SLE and right ventricular (25, 26). In our study, although no statistically causal relationship was established, a trend was observed indicating some degree of correlation between SLE and RVEDV (β = 0.006, *P* = 0.060). It is recognized that enlargement of the RV in SLE patients is typically attributed to pulmonary hypertension, which is caused by inflammation and vascular damage. However, a study by Deidda et al. demonstrated that subclinical RV dysfunction can be observed in SLE patients free of pulmonary hypertension using echocardiographic screening (25). Similarly, our study identified a trend towards statistical significance between SLE and increased RVEDV, suggesting that SLE could directly impact RV function. Several possible explanations may account for the lack of a positive result in our study. It is possible that in clinical practice, the use of medications and the presence of various comorbidities in patients contribute to the differences in outcomes, or that SLE induces changes in RV structure and function through alternative mechanisms, without a direct causal relationship between SLE and RV. Another possibility is the limited availability of data on RV within the GWAS data we included, which may explain the absence of positive findings (5).

As a chronic condition, SLE can have a persistent impact on cardiac and aortic structures and function, leading to gradual deterioration over time. These changes may eventually reach a critical threshold, triggering a transition from a subclinical to a symptomatic phase of heart disease. CMR, as an advanced tool, can detect these cardiac structural and functional changes at an earlier stage, making it valuable for early diagnosis and intervention. While previous research has demonstrated an association between SLE and cardiovascular disease, our findings may offer more direct evidence that SLE not only correlates with cardiovascular disease but may also directly impair cardiovascular function, which could be reflected in CMR measurements. Moreover, the CMR features associated with SLE could be further investigated as potential biomarkers for SLE-related cardiovascular damage. This could facilitate clinical monitoring and management of cardiovascular health in SLE patients, enabling earlier detection and intervention before clinical symptoms arise.

Our study showcases several strengths. First, MR was employed for the first time to explore the relationship between SLE and both cardiac structure and function using the latest CMR data. Second, MR effectively reduces the impact of residual confounding and reverse causality. By using sensitivity analyses and assessing the strength of IVs, the results were verified, thereby bolstering the robustness of the causal evidence. Third, since our findings predominantly rely on aggregated data from individuals of European descent, the potential bias due to population stratification is minimized.

Our study has some limitations. First, the scale and scope of genetic association studies on SLE have been limited, with constrained power and genomic coverage. To maximize power and coverage, we utilized the largest available GWAS of SLE. Second, as the prevalence and mortality of SLE exhibit variations across ethnicities, the exclusive inclusion of European participants in this MR analysis complicates the extrapolation of the potential causal relationship between SLE and CMR traits to other populations. Third, it is also worth noting that certain risk factors leading to different phenotypic outcomes, such as SLE and cardiac structure, may be influenced by environmental factors that cannot be fully explained by genetics. Our MR study can only address the genetically related components, and cannot fully account for the impact of environmental and non-genetic factors on these phenotypes. Lastly, the β value was relatively low and should be interpreted carefully.

In conclusion, we explored the influence of SLE traits on cardiac and aortic remodeling. Our findings suggest that SLE contributes to left ventricular remodeling by increasing LVSV, and both regional and global peak circumferential strain, while also leading to the reduction of regional radial strain. These findings may indicate a potential risk of cardiac function changes in SLE patients, aiding in the understanding of how SLE affects the cardiovascular system and providing guidance for cardiac examinations and disease prevention.

## Data Availability

Publicly available datasets were analyzed in this study. This data can be found here: the manuscript and Supplementary Materials contain all necessary data for evaluating its findings. For additional data related to this study, one can request it from the corresponding author upon reasonable request.
